# Educational interventions promoting modifiable lifestyle behaviours for cardiovascular disease in pre-registration nursing students: a scoping review

**DOI:** 10.1186/s12912-025-03611-x

**Published:** 2025-07-21

**Authors:** Laura Creighton, Gemma Caughers, Gary Mitchell, James McMahon, Lis Neubeck, Donna Fitzsimons

**Affiliations:** 1 School of Nursing & Midwifery, Queen’s University Belfast, Belfast, Northern Ireland, UK; 2 School of Health and Social Care, Edinburgh Napier University, Edinburgh, Scotland, UK

**Keywords:** Nurse education, Pre-registration nursing, Educational interventions, Modifiable lifestyle behaviours, Cardiovascular disease prevention, Blended learning, Smoking, Physical activity, Nutrition, Alcohol consumption

## Abstract

**Aims:**

Modifiable lifestyle behaviours, such as smoking and high alcohol consumption are more prevalent among pre-registration nursing students, than the general population. Embedding education on the impact of these risk factors for cardiovascular disease and motivating behaviour change is essential. This scoping review aims to investigate educational interventions promoting healthier lifestyle behaviours for cardiovascular disease prevention within the curriculum for pre-registration nursing students.

**Methods and results:**

The methodological framework for scoping reviews was used to identify literature on educational interventions for pre-registration nursing students, focusing on smoking, nutrition, physical activity, and alcohol consumption as key modifiable risk factors for cardiovascular disease. Following the PRISMA-ScR Checklist, 1631 records were screened using predefined inclusion and exclusion criteria, resulting in 29 records for data extraction and analysis. Interventions varied by region: North America (*N* = 7), Europe (*N* = 11), Asia (*N* = 8), and the Middle East (*N* = 3). Most studies targeted smoking cessation (*N* = 11), followed by nutrition (*N* = 6), alcohol (*N* = 3), or a combination of these (*N* = 9). Findings indicate that educational interventions improved knowledge, self-efficacy, and led to behaviour changes, particularly in smoking cessation.

**Conclusion:**

There are numerous educational interventions that aim to educate pre-registration nursing students about knowledge of modifiable lifestyle behaviours or supporting detrimental behaviour change in the clinical setting however, most interventions individually address a single behaviour such as smoking. This scoping review identifies that there is a paucity of educational interventions directly focused on cardiovascular disease prevention that encompass all four of the modifiable lifestyle behaviours of smoking, nutrition, physical activity and alcohol consumption in combination for pre-registration nursing students.

**Supplementary Information:**

The online version contains supplementary material available at 10.1186/s12912-025-03611-x.

## Background

Cardiovascular Diseases (CVDs) are the leading cause of death globally, most of which can be prevented by addressing modifiable behavioural risk factors [1]. Modifiable risk factors are those that can be changed to reduce the risk of CVD and are attributed to 57% of CVD prevalence in women and 53% in men over a 10-year period [2]. In 2021, 20.5 million deaths globally were related to CVDs, accounting for almost a third of worldwide deaths, with four out of five deaths occurring in low to middle income countries, highlighting large health inequalities and 80% of premature myocardial infarction and cerebrovascular accident being preventable [3]. Behavioural risk factors include tobacco use, alcohol consumption, diet quality, physical activity and sleep pattern, with a population-based longitudinal study suggesting that the hazard ratios for these remain similar across age but show increased risk with the addition of cardiometabolic conditions such as diabetes or hypertension [4]. It is paramount that CVD risk factors are addressed as a preventative health measure. The World Health Organisation (WHO) have identified four key behavioural based modifiable risk factors that increase risk of CVD as part of the umbrella term of Non-Communicable Diseases (NCDs), and these include tobacco use, physical inactivity, unhealthy diet and harmful use of alcohol [5]. 

Nurses are the largest healthcare discipline that manage risk factors and prevent risk for CVD for those in their care within primary, secondary, and tertiary settings [6]. They are well placed to provide education and support for the improvement of CVD-related modifiable lifestyle factors to reduce the risk of CVD. The available evidence indicates that the reception of health education among individuals accessing healthcare services, as well as the broader public, is influenced by the conduct of nurses themselves, highlighting the importance for nurses to actively role-model healthy lifestyle behaviours [7–9]. It may be postulated that due to their professional training and daily exposure to patients or clients afflicted with illnesses linked to CVD-related risk factors such as smoking and obesity, nurses would demonstrate lower engagement in such behaviours, however this is untrue. Internationally the prevalence of overweight and obesity amongst nurses was found to be between 55% and 79% ^10^, along with high levels of smoking [11], detrimental overconsumption of alcohol [11, 12], failure to meet physical activity guidelines of 150 min per week [11, 13] and poor nutritional habits., such as consuming 5 fruit and vegetables per day [11, 14–16]. This level of detrimental lifestyle behaviour is also mirrored in the pre-registration nursing student populations.

Pre-registration nursing students may develop unhealthy lifestyle habits due to the stress of transitioning to higher education, including being away from home, managing their own meals, and adapting to new shift patterns [17, 18]. A qualitative study in Australia involving nursing students [19] outlined the perception of healthy lifestyle behaviours as eating well, regular physical activity and limiting alcohol or nicotine. In spite of this a meta-analysis of smoking in nursing students worldwide showed a pooled prevalence of 26.6% [20], which is higher in comparison to the general population of 22.3% (36.7% men and 7.8% women) [21].

Another modifiable behaviour, alcohol consumption, showed 19.2% of female nursing students in a cohort in Ireland consumed more than the recommended weekly limit [22], while 10% of students in Scotland [23] and 4.6% of students in a study in Mexico are classified as high-risk consumers of alcohol [24]. In addition to smoking and alcohol, other modifiable behaviours in nursing students include decreased physical activity and poor nutrition. For example, Iran and Thailand found generally poor health promoting lifestyles amongst their nursing students with low levels of physical activity [25, 26], likewise, university students overall often struggle with poor diet and inadequate physical fitness.

Cross-sectional studies identified 69% of overweight or obesity amongst nursing students in Scotland [10], 50% of nursing students in a university in England [27], 34.7% Brazil [28], 57% United States of America [29], 61.3% Australia [30] and 79.1% South Africa [31]. Nursing students in the United kingdom were also spending up to six hours on average weekdays sedentary with a maximum of 20 h sedentary time per day [23]. A systematic review of university students showed a maximum sedentary time of 14.35 h [32] and although nursing students exceed this maximum it is the few and not the majority.

Nutritionally, 29% of students consumed less than the recommended five portions of fruit and vegetables with 40% consuming high fat and sugar food sources daily [27]. Nursing students often develop unhealthy habits early in their education, such as physical inactivity, stress, and poor nutrition, which are hard to change as they adjust to university and clinical environments [33, 34]. It is therefore important to understand what interventions can be implemented to provide education, motivation and facilitate behaviour change within the pre-registration nursing student population.

Identifying modifiable risk factors for cardiovascular diseases in university students early in their academic journey can facilitate targeted interventions to encourage healthier lifestyles and potentially reduce future health complications [35]. This is not only essential as nursing students are the future workforce and health promotors to those in their care, but personal lifestyle behaviours during their professional education can impact their risk either positively or negatively on their subsequent health status. Examining educational interventions that address the WHO’s four key modifiable lifestyle behaviours of (1) tobacco use, (2) physical inactivity, (3) unhealthy diet and (4) harmful use of alcohol [5] that are currently implemented in nursing curricula, including outcomes, will map the areas of successful behaviour change and gaps in education for pre-registration nursing students. This will allow development of future educational resources that can be implemented within pre-registration nursing programmes around the world to reduce CVD risk and improve modifiable lifestyle behaviour choices.

Therefore, the primary aim of this scoping review is to examine educational interventions that promote modifiable lifestyle behaviours for cardiovascular disease prevention which are embedded or delivered within pre-registration nursing education. This is achieved by three objectives:

### Objective 1

What lifestyle behaviours have been targeted in the educational intervention?

### Objective 2

What have the reported outcomes of the educational interventions been?

### Objective 3

What have been reported as the barriers or facilitators to implementation of the educational intervention?

## Methodology

A scoping review was conducted according to the accepted framework and reporting guidelines [36, 37]. The review methodology was chosen due to the breadth of literature being sought in the area of educational interventions related to cardiovascular disease as opposed to answering a specified question relating to confirming or refuting current practice such as the case in a systematic review [38]. A scoping review can be used to identify and map the available evidence in the literature, thus being able to help ascertain types of available evidence in a field alongside identifying and analyzing knowledge gaps [36, 38]. No timeframe was set for the literature included in the review to ensure that the breadth of records available for screening and therefore inclusion was not limited.

### Eligibility criteria

The eligibility criteria were developed using the Population, Concept, Context (PCC) framework as recommended by JBI [39] (Table 1).

**Table 1:** Eligibility criteria for scoping review

*Population*:	Nursing students enrolled in a pre-registration nursing course were included. Students enrolled in other disciplines or post-graduate nursing courses were excluded.
*Concept*:	Educational interventions that promote modifiable lifestyle behaviours for cardiovascular disease risk, focused on outcomes of either smoking, physical activity, nutrition or alcohol were included. Interventions that did not include any of the four modifiable behaviours were excluded.
*Context*:	The educational intervention must be delivered in or through a higher education institution such as a university or a college where professional nursing courses are taught. All other institutions were excluded.
*Types of evidence source*:	Qualitative, quantitative or mixed-methods studies in English language. Grey literature and literature reviews identified had the citations hand searched for further eligible articles. Conference abstracts, editorials, opinion pieces, newsletters and blogs were excluded due to intervention description being essential to mapping the evidence. There was no timeframe specified for study inclusion.

### Identifying relevant studies

Following preliminary searches of the electronic databases and in consultation with a subject librarian, search terms were finalised. The search occurred in October 2024.

A comprehensive search was undertaken of; CINAHL, Medline, Embase, Scopus, ERIC and British Education Index. The search terms and search strategy for CINAHL are included (Table 2). The grey literature search involved the hand searching of relevant cardiac health and charity websites, for example World Health Organisation (WHO), European Society of Cardiology (ESC), American Heart Association and Australian Cardiovascular Health and Rehabilitation Association (ACRA). Search results were imported to Covidence https://www.covidence.org/, a screening and data extraction tool that streamlines the process of reviews, ensuring efficiency and tracking of the process. The titles and abstracts of all records were independently screened by two individuals: LC (100%), JM (50%) and GC (50%). Conflicts were managed through an individual not involved in screening (GM). This process was repeated for round two screening.

**Table 2:** Search terms and example search strategy from CINAHL

Modifiable Lifestyle Behaviours	Intervention	Pre-registration nursing Students
MESH headings	MESH headings	MESH Headings
Cardiovascular diseases/OR	Learning/OR	Students, nursing/OR
Heart disease risk factors/OR	Educational Technology/OR	Education, nursing, baccalaureate/OR
Health behavior/OR	Gamification/OR	
Smoking/OR		Keyword
Exercise/OR	Keywords	Student nurs*/OR
Diet/OR	Educational intervention*/OR	Undergraduate nursing studen*/OR
Alcohol drinking/OR	Training program*/OR	Pre-registration nursing student*/AND
	educational resource*/OR	
Keywords	Pedagogy/OR	
Cardiovascular risk factors/OR	Training/OR	
Modifiable behav*/OR	E-learning/OR	
Behav* change/OR	Online education/OR	
Health behav*/OR	Blended learning/OR	
Physical activity/OR	Curriculum/OR	
Nutrition/OR	Digital education/OR	
Modifiable risk factor*/AND	Serious educational game/OR	

### Charting the data

Data extraction was undertaken using the data extraction template (Table 3) modified using JBI framework Appendix 11.1 [39]. A pilot data extraction of a 20% sample full text articles identified for inclusion in the scoping review was undertaken independently by LC and JM. The rationale for a scoping review is not to produce a critically appraised answer to a particular question but to provide an overview or map of the evidence [38]. Therefore, it is not a required step of a scoping review to critically appraise the included articles but to still maintain a rigorous approach by following an ‘a priori’ protocol [39]. In this review, methodological quality and risk of bias was done on the included empirical research articles using the Mixed Methods Appraisal Tool (MMAT) to identify high, medium and low quality of evidence [40]. This was alongside an ‘a priori protocol’ registered on Open Science Framework https://osf.io/6nf2w.

**Table 3:** Data extraction template

Scoping review details		
**Scoping Review Title**:	Educational interventions promoting modifiable lifestyle behaviours for cardiovascular disease in pre-registration nursing students: a scoping review.	
**Scoping review Primary question**:	What educational interventions have been implemented for pre-registration nursing students that promote modifiable lifestyle behaviours for cardiovascular disease?	
**Scoping review sub-question(s)**:	1) What lifestyle behaviors have been targeted in the educational intervention? 2) What have been reported as the barriers or facilitators to sustain long-term modifiable behavior change for pre-registration nursing students?	
**Title of article**:		
**Article ID**: (surname of 1st author, year of publication and letter if more than one year e.g. Creighton 2023b)		
**Country of Origin**:		
	(please state)	Source (page number)
**Eligibility criteria**		
**Population**: Adult (aged over 18 years old) Pre-registration nursing student		
**Concept**: Educational intervention focusing on a modifiable lifestyle behaviour for cardiovascular disease:- 1. Physical activity 2. Smoking 3. Nutrition 4. Alcohol		
**Context**: Educational intervention delivered in a higher education institution		
**Type of Evidence Source**:		
**Aim**:		
**Population and sample size**: Age Gender Sample size		
**Methodology**:		
**Intervention Type**: -how was the intervention delivered? -what was the duration of the intervention? -who was the intervention delivered by?		
**Outcomes of the intervention**- How was this measured? What outcomes were focused on?		
**Longevity of intervention** Were the long term effects of the intervention mesured? How was this measured? What was the length of time?		
**Barriers and facilitators to sustained behaviour change?** Barriers described? Facilitators described? Further recommendations noted		

### Critical appraisal

The MMAT [40] scores are out of five for qualitative and quantitative studies and 15 for mixed-methods. Of the 29 records included 16 scored the maximum of 5/5, six scored 4/5, three scored 3/5 and one score 2/5. Of the three mixed methods scores were 11/15, 13/15 and 15/15 (Supplementary file 1). This indicates that majority of records included are of a high quality, however, those that were assessed to be of lower quality were not excluded.

### Collating, summarising and reporting of results

A PRISMA extension for Scoping Reviews explanation and elaboration (PRISMA-ScR) [39] flowchart was developed during the screening process for transparency. Analysis of the evidence was carried out using the three-step framework [41]. Quantitative data was analysed using simple frequency counts of concept occurrence, characteristics and populations [42]. Qualitative data was analysed using basic content analysis allowing results to be coded and mapped into particular categories aligned with the scoping review questions and sub-questions, as they occurred. Themes identified from analysis are descriptively presented.

## Results

**Fig. 1:**
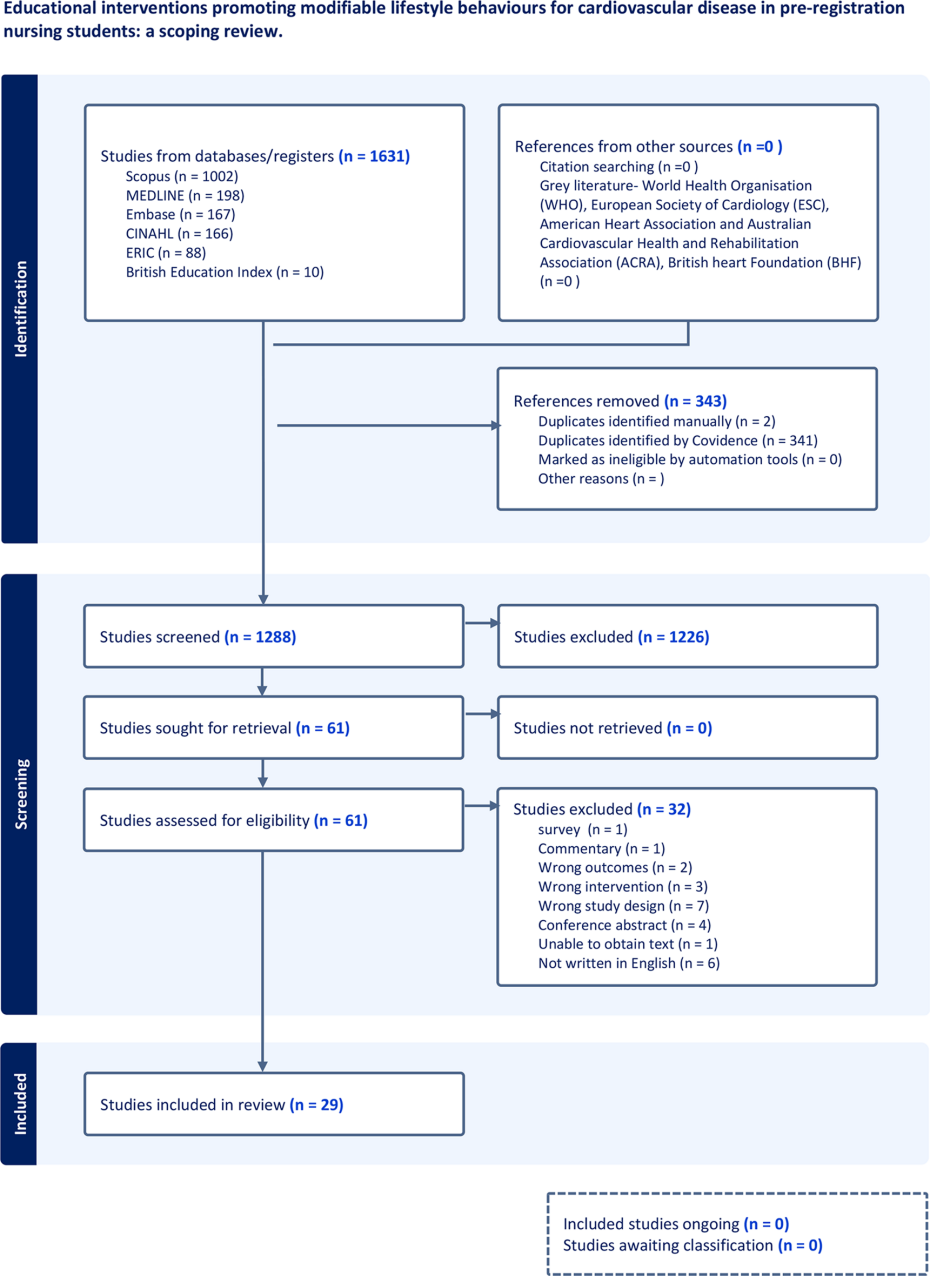
PRISMA-ScR flowchart

A total of 1631 records were identified, with 343 duplicates removed prior to the screening process. On conclusion of screening, 29 records were included in the scoping review (Fig. 1).

### Country of origin

**Fig. 2:**
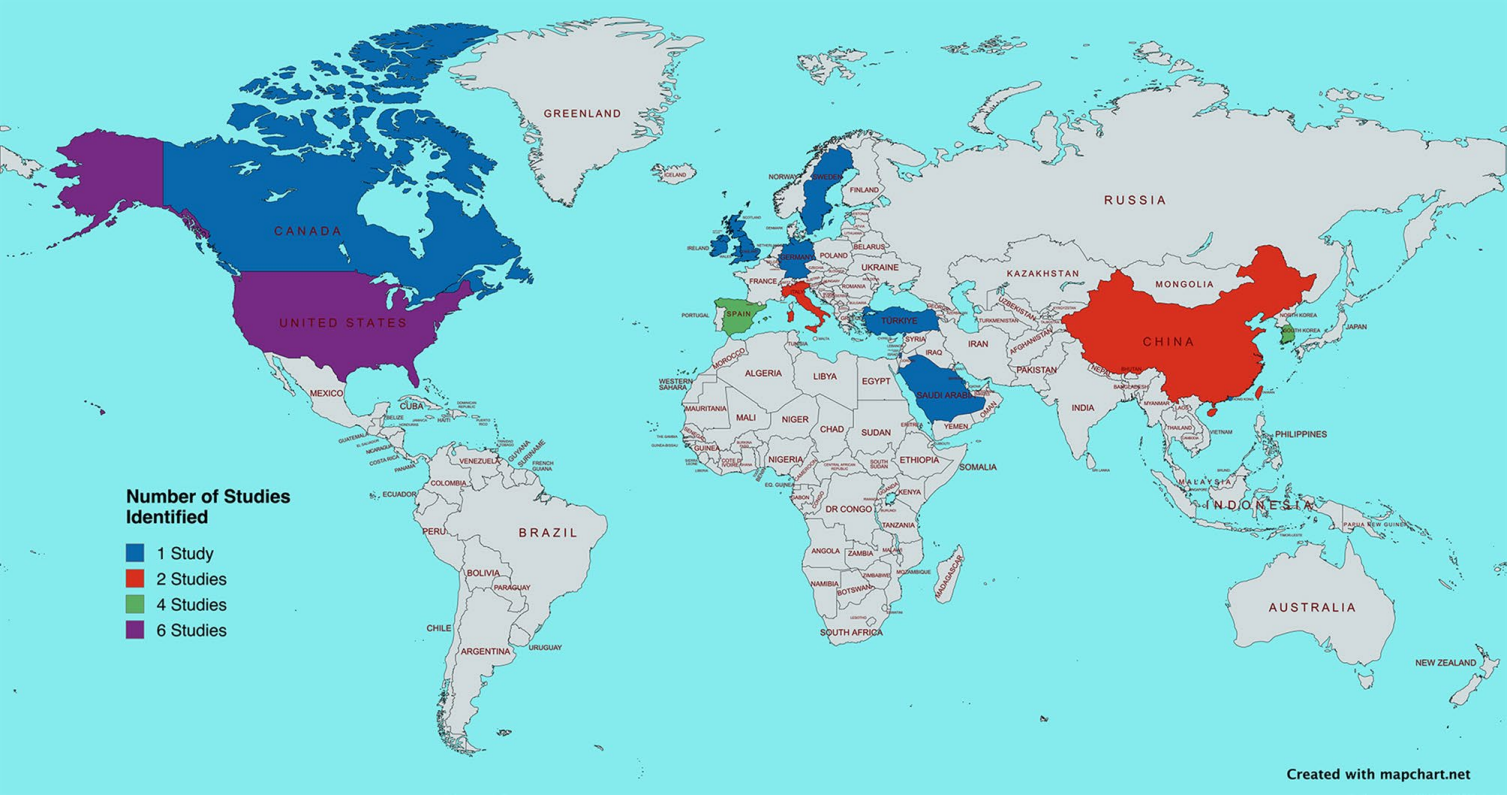
World map presentation of included record origin

The majority of studies were undertaken in Europe (*n* = 11) [43–53], with an international breadth of studies across North America (*n* = 7) [54–60] Asia (*n* = 8) [61–68] and the Middle East (*n* = 3) [69–71] as represented by the map (Fig. 2).

### Modifiable behaviour delivered in the educational intervention

Smoking was the most frequently occurring modifiable behaviour delivered as an educational intervention, with 11 studies describing solely smoking cessation interventions [48, 57–60, 62–64, 66, 67, 71]. However, nine studies were comprised of a mix of modifiable behaviours of which smoking was included in five [45, 49, 55, 61, 68]. Physical activity did not appear as the sole focus of any of the educational interventions but was combined with other behaviours such as nutrition [64]. Nutrition was the focus of six studies [44, 46, 47, 54, 69, 70] with alcohol consumption featuring in three studies [44, 51, 53]. Only one study delivered an educational intervention that combined all four of the modifiable behaviours but this was not specific to CVD prevention [49]. The intervention type and outcomes of this review are presented based on intervention focus to enhance clarity and coherence. By organising the findings according to specific behaviour change strategies, such as smoking cessation, alcohol reduction, physical activity promotion, and dietary improvements, we ensure that each intervention’s effectiveness is evaluated within its relevant context within this review.

### Educational interventions on smoking

Eleven studies were included: nine were quasi-experimental design [48, 59, 60, 62–64, 66, 67, 71] one was a randomised controlled trial [58] and one qualitative study using focus groups [57] (Table 4).

**Table 4:** Data extraction findings for educational interventions on smoking

Author, year	Country	Journal	Sample Size	Aim	Methodology	Type of Intervention	Barriers (B) and Facilitators (F)	Key Findings
Shin and Lee, 2023	South Korea	Healthcare	*N* = 67	To contribute to the development of smoking cessation counseling educational content that can be used for undergraduate nursing curricula.	Quasi-experimental study design	Multi-modal dependent on group allocated. Lecture, online video and peer role-play.	**B**) Nursing students who smoke are less likely to be perceived as role models for patient smoking cessation than nonsmoking nursing students. **F**) Role play as a method of smoking cessation delivery had the highest increase in attitudes towards smoking, therefore active learning methods are required alongside passive	Attitude, self-efficacy and intention to deliver smoking cessation intervention increased across all 3 groups.
Lee and Yunhee, 2022	South Korea	Korean Academic Society of Nursing Education	*N* = 52	To apply a flipped learning-based quit smoking intervention education program for nursing students and to verify its effects	Quasi-experimental study with a pre-posttest non-equivalent control group	Real time video classes via Zoom and face to face classes	**B**) The length of time between pre-class learning and in-class activities. **F**) Convenient for students to take online lectures at any time and class activities were more active.	The intervention group had a statistically significant difference- showing a greater degree of beliefs about the health benefits of quitting smoking and attitudes towards quitting.
Zhang et al., 2021	China	Tobacco Induced Diseases	*N* = 289 all 3 parts of questionnaire	To assess the impact on the knowledge, attitudes and self-efficacy, as well as the behaviours of nursing students for assisting patients in quitting smoking	A one-group study design	2 h didactic lecture, 2 h online learning and 2 h simulation	**B**) Lack of knowledge of how to engage with patients on smoking cessation. **F**) Blended learning delivery of the resource Scenarios used in the simulation case studies were reflective of what is encountered by the students in real life clinical practice	Significant improvement in knowledge of tobacco cessation post intervention, self-efficacy and delivery of interventions whilst in clinical practicum.
La Torre et al., 2019	Italy	Journal of Preventative Medicine and Hygiene	*N* = 365	1) To evaluate the effectiveness of the course to change smoking status of students attending the course 2) To do a quantitative and qualitative analysis of the course through the comments and opinions that students expressed about it.	An experimental study, a pre-post design with a single group.	Online	**B**) Students commented on the length of the course as a barrier. Lack of contact with university faculty due to online nature of the course. **F**) Online delivery is convenient. Good content and essential message of smoking cessation.	161 (44.1%) students were classified as smokers, decreased to 141 post the intervention (38.9%). 11.8% stopped smoking and 7.5% tried to stop. Nursing students were the most prevalent group of smokers.
Hamedah et al., 2018	Bahrain	BMC Medical Education	*N* = 121 nursing students	To assess the impact of an educational intervention on the health of professional students’ knowledge about Waterpipe Tobacco Smoking and the health effects associated with it.	Quasi experimental study	Video and a lecture	**B**) Lack of embedded teaching about WTS in the nursing curriculum. Students were found to have gained their information most frequently from social media on WTS so this intervention ensures expert teaching	There was an increase of 12.9% in intention to quit smoking following the intervention.
Choi et al., 2018	South Korea	Osong Public Health and Research Perspectives	*N* = 44	To evaluate whether a smoking cessation intervention education program based on blended learning, was effective in improving nursing students perceived competence and motivation	A quasi experimental design, using a pre-post test control group	Intervention group- E-learning program and face to face classroom teaching	**B**) Lack of knowledge and inexperience concerning performance of a smoking cessation intervention	Intervention group showed significant improvements in nursing students autonomous motivation, controlled motivation and perceived competence in performing a smoking cessation interventions
Schwindt et al., 2016	USA	Journal of Nursing Education	*N* = 134	(1) Students will report a greater increase in perceived competence to engage in tobacco cessation interventions with patients with mental illness who have received an ADT based education programme in addition to receiving standard tobacco content only (2) Students will report a greater increase in intrinsic motivation to engage in tobacco cessation interventions	Randomised control group design	Intervention group online and face to face	**F**) Students may have developed the values and beliefs of their mentors/faculty as the importance of the training and study itself identified that smoking cessation was important	The intervention group perceived themselves as significantly more competent to engage in tobacco cessation training. Motivation was not significantly different between groups.
Schwindt et al., 2016	USA	Archives of Psychiatric Nursing	*N* = 7	Explore student perceptions of the tobacco education intervention	Qualitative audiotaped focus groups	Online and face to face	None cited	3 themes developed 1) Gaining new knowledge and applying to practice, 2) Using a non-judgmental and empathetic approach to care and 3) Recommendations for curricular change.
Shishani et al., 2013	USA	Nurse Education Today	*N* = 110	To examine the effect of an evidence-based smoking cessation educational program on senior nursing students’ perceptions and self-confidence in their ability to help smokers quit.	Pre-post test survey	Online and practical face to face simulation	**B**) The use of the online program was not monitored and so could not be certain all students completed program. **F**) Students find online learning convenient and flexible.	Significant gain in knowledge with 70% students expecting to use the program in their work with patients. Significance in 4/5 aspects of the 5As. Lack of confidence was identified in asking patients if they were smokers.
Sohn et al., 2012	South Korea	Nurse Education Today	*N* = 21	(1) to describe nursing students’ experience, attitude and perceived barriers of smoking cessation intervention (2) to describe a simulation-based training of smoking cessation intervention (3) to evaluate its effectiveness on nursing students self-efficacy in performing smoking cessation intervention	One group quasi-experimental study	Face to face lecture and simulation	**B**) Lack of skills and knowledge of smoking cessation, nurses own smoking and patients lack of interest in smoking cessation. Smoking in South Korea is considered part of male culture and is often a hard topic for nurses (who are mostly female) to discuss with male patients. **F**) Teaching method of simulation- allows building communication skills in a safe environment and allows facilitator to highly standardise the content.	Self- efficacy significantly improved post intervention. 20% students had the opportunity to practice smoking intervention in practice despite encoutering patients who smoke on a weekly basis
Butler et al., 2009	USA	Journal of Nursing Education	*N* = 178	1) Pilot test the intervention and the survey instrument (cohort 1) 2) whether participation in the program would increase skill, confidence, knowledge and perception of how active a role nursing should take in promoting cessation 3) would 6 h participation have greater effect than 2 h 4) examine the relationship between participant smoking status and study outcomes	A quasi-experimental design with two cohorts of nursing students	Didactic lecture for all students and interactive practice intervention group	**B**) 6 h session perceived as ‘too long’ by students. Smoking as a behaviour amongst students may impede their confidence in delivering smoking cessation interventions to patients **F**) More hands on activities for learning.	Students improved significantly in skill, confidence and knowledge in boths groups showing no difference in the 6 h–2 h sessions.

#### Objective 1: Intervention type

All eleven interventions focused on smoking cessation education and employed a mix of passive and active learning methods. Six interventions integrated various approaches: for instance, one study included pre-learning followed by lectures, online video groups, or case-based peer role plays [62]. Another study combined two hours of didactic lectures, two hours of online learning, and two hours of scenario simulation [64]. Some studies used online education followed by simulations or case-based activities [59, 67]. Other interventions included a standard one-hour lecture with additional interactive classes [58] or ten sessions of e-learning and classroom activities [66]. A flipped classroom approach with pre-learning and in-class activities was used in one study [63]. The longest intervention spanned five days with lectures and debates [48], while a study focused on Waterpipe Tobacco Smoking with a brief video and lecture [71]. Additionally, a study compared a six-hour comprehensive program with a shorter two-hour version [60].

#### Objective 2: outcomes

In the smoking cessation studies, four main outcomes were identified, and these were self-efficacy, attitudes, knowledge and behavioural change.

Self-efficacy showed significant improvement across several studies. A statistically significant difference (*p* < 0.001) was noted in self-efficacy post-intervention for all participants receiving education about smoking cessation (*n* = 67), with the greatest increase observed in those receiving online video-based education [62]. Another study found significant improvements in self-efficacy across five key areas, including advising patients and helping them set a quit date when undertaking case-based activities [67]. Further, self-efficacy was significantly higher in a ‘flipped classroom’ intervention compared to the control group (*p* = 0.003) [63]. Similarly, significant gains were reported across all timepoints in a sample of 286 nursing students following simulation [64]. 

Attitudes towards smoking cessation interventions also improved. Four studies utilising validated questionnaires found that nursing students developed a more positive attitude towards smoking cessation and enhanced self-efficacy following educational interventions. This improvement was consistent across studies that employed didactic lectures combined with simulation or peer role play [62–64, 67]. 

Knowledge about smoking cessation also increased significantly in four studies, as indicated by improved scores on knowledge questionnaires post-intervention [48, 60, 64, 71]. One focus group study also revealed that students gained new knowledge, applied it in practice, and integrated it into the curriculum [57]. Finally, behaviour change was evidenced in just one study, which reported a 5.2% reduction in smoking prevalence among participants in a follow-up survey conducted four months after the intervention [48]. 

### Educational interventions on nutrition

Six studies focused on interventions related to nutrition: four of these were cross-sectional studies [43, 46, 54, 70], one was a quasi-experimental study [69] and the other a mixed-methods study [47] (Table 5).

**Table 5:** Data extraction findings of educational interventions on nutrition

Author, year	Country	Journal	Sample Size	Aim	Methodology	Type of Intervention	Barriers (B) and Facilitators (F)	Key Findings
Mancin et al., 2024	Italy	Teaching and Learning in Nursing	*N* = 66	To evaluate the nutritional knowledge of first-year nursing students before and after the administration of a clinical nutrition learning module using active teaching methodologies	A cross-sectional observational study	4 h face to face teaching and 2 h clinical discussions using interactive assessment systems	**F**) The use of an Audience Response System (ARS) may encourage students who would be reluctant to speak out in class to participate as the answers given are anonymised.	There was an increase in nutritional knowledge of the sample of students considered.
Thang et al., 2023	USA	Nutrients	*N* = 75 of which *N* = 37 nursing students	To investigate the impact of the Upstream Obesity Solutions (UOS) program on the knowledge acquisition, attitudinal shifts and practical application of healthcare trainees pertaining to nutrition education, lifestyle habits and culinary skills.	Cross-sectional descriptive study	Blended learning methods Lectures face to face, self study and kitchen experiences.	**F**) Interactions with interdisciplinary faculty members may have contributed to observed self-perceived improvements in cooking skills and nutrition literacy among learners. The variety of learning methodologies the program comprised of.	Confidence in knowledge about obesity and confidence in counselling about obesity significantly improved. General knowledge about obesity significantly improved for nursing students. Nursing students had statistically significant improvement in days consuming at least 5 servings of fruit and vegetables post intervention.
Zagamir et al., 2023	Kingdom of Saudi Arabia	Libyan Journal of Medicine	*N* = 250	Evaluate the efficiency of an intervention study on nursing students’ knowledge and practices regarding nutrition and dietary habits.	Quasi-experimental pre-post design without a control group	4 h training per group- mode of delivery not specified	**B**) How students get their information on nutrition is a barrier as the prevalence is social media whereby the information may not be correct or reliable. Nursing as a course can be busy and stressful and students can adopt poor dietary habits as away from the home environment and physical exercise may decrease due to schedules. **F**) Increasing knowledge and engaging in positive behaviours as an undergraduate will be beneficial for students as they move through adulthood as poor behaviours have a likelihood to impact long tern future health.	Main sources of knowledge and information- 64% social media, 18% TV and radio, 8% family and friends, 7% health campaigns and 3% health care providers. This highlights the critical need for reliable information sources and the significant impact that social media wields in respect of information sharing. Considerable increase in the total knowledge score on nutrition and food habits post intervention.
Natour and Al-Tell, 2022	State of Palestine	Health Education Journal	*N* = 106	How students received MYPlate, how they applied it, and whether changes in their dietary patterns based on the website were observable.	A cross-sectional study	As part of a compulsory course called pre-clinical nutrition. Method of delivery unclear.	**B**) MYPlate is a website so may not be accessible to all students. **F**) Students who already engage in physical activity, eat vegetables or are from higher income families display use of MYPlate and more nutritional awareness.	88.7% students were aware of the MYPlate application. 61.3% had used the app in their diet planning and found this easy to do. 72% had used it to plan their diet or find the number of calories they needed. 40% ate fruit and vegetables between meals. Use of MYPlate was not related to nutritional problems or the desire to increase or decrease weight.
Rosa- Castillo et al., 2022	Spain	Nurse Education Today	*N* = 106	Improving students learning process and motivation	Cross-sectional, descriptive, observational study	Online via Instagram social media platform	**B**) Planning, preparing and executing and tracking the Instagram channel is time consuming for the staff involved. Risk of time management, social media addiction and exposure to negative content for students on social media. **F**) Accessible for students in their own chosen time. Once set up for staff can be adapted easily for use again.	71.7% of students agreed that participating helped them learn the content and that participating helped them consolidate what they had learned in class.
Holmberg et al., 2021	Sweden	Nurse Education Today	*N* = 161	To describe undergraduate nursing students’ learning outcomes and important elements for their learning conducting a one-day food recording with a subsequent seminar.	A cross-sectional and descriptive student evaluation (mixed-methods, questionnaire and open ended qualitative complementary data)	An independent activity and consolidated as a face to face webinar.	**B**) The food dairy was a voluntary aspect of a mandatory course, therefore not all students participated. **F**) Students liked the active learning aspect. This was student led and experiential.	Increased understanding of the nutritional recommendations. Becoming more aware of their general dietary health and nutrients in different foods. Students had a higher pass rate of 96% in their examinations who attended the seminars in comparison to 71% who did not attend.

#### Objective 1: Intervention type

Four studies used various teaching methodologies to enhance learning. One intervention included a mandatory week-long nutrition course with lectures and an exam, supplemented by a voluntary in-person workshop on using a personal food diary [47]. Another involved four hours of nutrition and dietary habits teaching using interactive lectures, group discussions, and demonstrations [69]. A 10-week course on Health Promotion of Nutrition combined in-class lectures, a self-study module, and teaching kitchens for culinary skills [54], while a six-hour program over two days focused on clinical nutrition with didactic lessons and a two-hour clinical case workshop using an interactive audience response system [43]. Two studies used technology enhanced learning as their approach and this included a four-week Instagram-based game on nutrition, featuring meal photos, team challenges, and gamified feedback [46]. Additionally, online training using the MyPlate website was provided for nursing, medicine, and pharmacy students [70].

#### Objective 2: Outcomes

In the nutrition-based studies, three main outcomes were identified, and these were knowledge, feasibility and satisfaction.

All four studies identify an improvement in knowledge post the educational intervention on nutrition. Knowledge was assessed in one study using a questionnaire that underwent face validity and indicated an increased understanding of nutritional recommendations, awareness of general dietary health and nutrients in different foods in a sample of 161 nursing students [47]. This was supplemented by free-text data that indicated students preferred the active learning of conducting a food diary over the traditional didactic lectures, as it allowed comparison or their own records with nutritional guidelines [47]. Similarly, a study of 37 nursing students found that general knowledge about obesity was significantly improved (*p <* 0.05) following the intervention and evaluated using a non-validated questionnaire [54]. This was echoed by findings from a sample of 250 nursing students, where a significant difference in knowledge of nutrition and eating habits post-intervention was identified using a non-validated questionnaire that underwent face validity prior to use [69]. A validated questionnaire to evaluate the level of learning of basic clinical nutrition issues was utilised in another study sample of 63 nursing students and demonstrated a statistically significant increase (*p* < 0.001) in nutritional knowledge post-intervention [43]. 

Use of a social media platform for nutrition gamification showed 71.7% students (*n* = 106) in agreement that participating had helped them learn the class content and that gamification should be used more in other subjects [46]. 

### Educational interventions on alcohol

Regarding educational interventions that considered alcohol consumption, there were three studies that were included in this review. Two of these used a mixed methods design [44, 51] while another used a quasi-experimental design [53] (Table 6).

**Table 6:** Data extraction findings of educational interventions on alcohol

Author, year	Country	Journal	Sample Size	Aim	Methodology	Type of Intervention	Barriers (B) and Facilitators (F)	Key Findings
Lavilla- Gracia et al., 2023	Spain	Nurse Education Today	*N* = 21	(1) Design, implement and evaluate a motivational interviewing training course for alcohol misuse in an undergraduate nursing curriculum (2) explore students’ experiences with the course	Mixed-methods approach Descriptive comparative quantitative design and qualitative focus group interviews	Didactic lectures and practical classes	**F**) Novelty of the innovative delivery of the course may have increased students motivation to learn.	Knowledge- 80% students passed the exam with an average grade of 8.39/10. Focus groups identified 3 themes- (1) learning atmosphere, (2) Module methodologies (3) students self-perception of confidence.
O’May et al., 2016	Scotland	Nurse Education in Practice	*N* = 64 nursing students	1) document knowledge and understanding of final year nursing and occupational therapy students in relation to alcohol misuse and alcohol intervention before, immediately following and 3 months after, attendance at an ABI workshop 2) explore students now qualified practitioners retrospective perceptions of the workshop, its content and relevance to their practice	A mixed-method prospective cohort design	Delivered as a ‘hands on’ interactive workshop	**B**) Students considered themselves as health care professionals that should be using ABIs however (61–69%) had the personal opinion that people should have the right to use alcohol as they wish within the confines of their own home, creating an attitudinal dissonance. This could impact on their attitudes to ABI as practitioners, when confronted with having to ask questions relating to alcohol. **F**) ABI was received as an important area of knowledge to be acquired	On follow up of the students since qualifying at the 3 month point no-one had used an ABI in practice. Participants gave reasons for not doing so as lack of confidence and experience.
Rabanales Sotos et al., 2015	Spain	Nurse Education Today	*N* = 1060	1) To ascertain nursing students’ level of knowledge about preventative activities targeting excessive alcohol consumption 2) To assess their skills- acquired through self-assessment of their own alcohol consumption	Before-after intervention study	Teaching workshop (mode of delivery unclear)	**B**) Lack of knowledge of what hazardous drinking is and how to use the tools **F**) Active learning through self-assessment in using the tool they would use in clinical practice with placement.	Pre- intervention- 24.1% knew the concept of a hazardous drinker, increased to 95.8% post. The prevalence of hazardous drinking in the student sample as a whole was identified as 43.4%.

#### Objective 1: Intervention type

Three interventions were identified, two of which were teaching-focused while the third was a longer, multifaceted intervention [44]. These interventions varied significantly in duration, lasting 1 h [53], 1 day [51], and 12 weeks [44], respectively. One study used a one-hour workshop on alcohol prevention, where nursing students practiced self-administering tools commonly used to detect hazardous consumption of alcohol in clinical settings. This workshop helped familiarise students with these assessment tools [53]. Another intervention involved a mandatory one-day face-to-face workshop on alcohol brief intervention training delivered by national trainers [51]. The longest intervention spanned 12 weeks of blended learning, including didactic lectures, video visualisations, and role-play exercises across modules on motivational interviewing and alcohol consumption in university students, delivered in two-hour sessions per week [44]. 

#### Objective 2: Outcomes

Of the studies that focused on alcohol consumption within this review, only one outcome was reported, and this related to knowledge. All three studies focused on the outcome of assessment of knowledge. One study utilised a mixed-methods approach, combining an exam, video scenario recordings, and focus groups to evaluate motivational interviewing skills and alcohol-related concepts [44]. Results of this study showed that 80% of the 21 nursing students passed the knowledge exam, with their skill acquisition improving from 15.7/27 to 19/27 after feedback and practical workshops. The second mixed methods study used an internally produced pre/post-questionnaire to determine knowledge of public health messages, with focus groups to explore learning and perceptions of barriers and facilitators to education across two cohorts [51]. The study evidenced that two thirds of students had correct answers at baseline but there was no significant increase at three month follow up. Similarly, a large study of 1,060 nursing students showed poor baseline knowledge of hazardous drinking. However, after one hour of training and self-assessment, knowledge improved significantly: correct answers on hazardous consumption limits increased from 24.1 to 95.8%, and quantification of alcohol consumption rose from 3.1–92.5%.^53^

### Educational interventions with mixed modifiable behaviours

Within this review, there were nine studies which considered a mixture of the modifiable behaviours for CVD. Of these, seven quasi-experimental [45, 49, 50, 53, 56, 61, 68], one was a randomised controlled trial [55] and one was an explorative study [65] (Table 7).

**Table 7:** Data extraction findings of educational interventions with mixed modifiable behaviours

Author, year	Country	Journal	Sample Size	Aim	Methodology	Modiable Behaviours	Type of Intervention	Barriers (B) and Facilitators (F)	Key Findings
Dong et al., 2023	China	Nurse Education today	*N* = 87	Evaluate the delivery and the effectiveness of online cardiovascular health behavior modification training	Non-equivalent, quasi-experimental design to compare pre-and post-test results	Smoking, nutrition and physical activity	Online	**B**) Online lecture may be too passive in comparison to the addition of a practical role play. **F**) Online learning is not constrained by time or space.	Health Education competency and clinical decision making perception score were both higher post education sessions in both the control and intervention groups. Intervention group showed significantly higher difference in all aspects compared to the control group.
Perez-Rivas et al., 2023	Spain	BMC Nursing	*N* = 2187	To determine the effectiveness of a learning strategy based on learning by doing and grounded in the nursing process, on the improvement of lifestyle in students of nursing	Quasi-experimental intervention over an 11 year period. Repeated measures carried out 3 months after the intervention	Smoking and Obesity	3 h seminar with mandatory attendance 3 month intervention period	None cited	949 students were identified as having at least 1 risk factor 432 (19.8%) were smokers 517 were overweight (23.6%), 108 obese (4.9%) Reduction of smoking was the most successful intervention with 75 students stopping or reducing their smoking.
Chang and Chen, 2020	Taiwan	The Journal of Nursing Research	*N* = 57	To explore the experiences and facilitators affecting health-promoting learning with reflective teaching in nursing students	An explorative study that used qualitative data analysis.	Physical Activity, Nutrition and Hydration	9 weeks delivered classroom teaching and 4 week reflective activity for the students	**B**) Easier said than done Forming a habit Goals that are too ambitious Lack of motivation or support **F**) Students supporting one another Use of technology to support and motivate The joy in completing the behaviour Adjusting the goals after one week to see if there were achievable and make incremental goals	Regular exercise was the most often cited, followed by a balanced and healthy diet and then daily water intake. 3 themes developed (1) Easier said than done (2) Compromise and adjustment (3) Continuation of health Behaviours
Fontaine and Cossette, 2021	Canada	Nurse Education Today	*N* = 67	To evaluate an asynchronous adaptive e-learning program based on the Theory of Planned Behaviour, Cognitive Load Theory and the concept of engagement compared to a knowledge based standardised e-learning program to increase nurses’ and nursing students’ intentions to provide brief counselling.	A two group, single blind, randomised controlled trial	Smoking and Nutrition	Asynchronous e-learning	**B**) The experimental group program entailed clinical staff outlining how to overcome the barriers of brief counselling and this may have drawn attention to them. Participation in the e-learning was not mandatory Less clinical experience of nursing students may impede provision of brief counselling. **F**) Asynchronous	Statistically significant increase from baseline to follow up scores for intention to provide brief counselling in both groups. Both e-learning programs influenced beliefs and behavioural control similarly regardless of the additional adaptive content.
Nevins et al., 2018	USA	Journal of Holistic Nursing	*N* = 21 pre-survey and 12 post survey	To explore strategies used to address the exercise and hydration needs of baccalaureate nursing students.	A descriptive, quantitative design	Physical Activity and Hydration	Intervention strategies were presented through e-mail and flyers to students	**B**) Academic workload, personal life demands, varied health care goals of students and inconsistent peer leader or faculty support. **F**) Increased awareness may be achieved through use of familiar, popular devices such as water bottles or pedometers.	Positive trend toward exercise and a statistically significant increase in water consumption from pre-post survey.
Wills and Kelly, 2017	United Kingdom	Nurse Education today	*N* = 244 first time point *N* = 302 s time point *N* = 96 final wave 41 participants completed all 3 time points	Explore the health of student nurses, whether training effects their health over time and what might help them to maintain a healthier lifestyle.	Before and after study with a single cohort of students	Physical activity, smoking, nutrition and alcohol	3 different interventions 1) 1 h educational session 2) Pulse accelerometers and a virtual learning environment to log steps 3) An online wellness tool for personal goal setting	**B**) Project was voluntary so those who participated may be more health conscious or healthier at baseline hence the participation. Clinical placement and the stress of adapting to shiftwork or the role of the nurse. **F**) Incorporating the intervention into the curriculum. Healthy environments at university- environmental supports such as subsidised gyms and a healthy eating canteen.	No significant change in self-rated health amongst participants over the course of the study. 45% of participants at point 3 reported that their health had got worse since they started their training. The effects of placements on participants health in relation to stress, lack of time and irregular routine lessoned over the course of study. A decline in smoking- 18% point 1 and 12% point 3 was recorded.
Kara, 2015	Turkey	Nurse Education today	*N* = 108	To examine the long term effect of the educational intervention on improving the health behaviours of Turkish female baccalaureate students	A quasi-experimental study with one group pre and post test and a 3 year follow up.	Physical Activity and nutrition	Theoretical courses and practical training	**F**) Attendance at the 4 week course was mandatory	The educational intervention showed a significant effect on the overall health behaviours of the nursing students. Students showed some improvements in the exercise, nutrition and stress management scores at post intervention.
McSharry and Timmins, 2016	Ireland	Nurse Education today	*N* = 110 total *N* = 49 nursing students	To evaluate the impact of the inclusion of a newly developed health and wellbeing course	A quasi-experimental pre-post test quantitative study	Physical Activity and Nutrition	Face to face theoretical and practical workshops	**B**) Can be difficult to receive nutrition advice from a nurse who themselves is overweight. Nursing students are disadvantaged in being able to engage with university provided exercise and health facilities. **F**) Developing peer, classroom and online support structures that support students during a turbulent time.	Study showed that all students gained weight in their first year at university. Intervention group (Nursing students) showed significantly significant improvement in psychological wellbeing between pre and post intervention.
Hsiao et al., 2005	Taiwan	Public Health Nursing	*N* = 65	To develop a teaching course on health promotion for nursing students and examine the effects of the course	Pre-test post-test questionnaire with a content analysis on the written reports of the students	Physical Activity, Nutrition and Smoking	Classroom lectures	**B**) Students focused on the physical aspects only as opposed to a holistic approach which is found in nursing care. **F**) Report recording behaviour change added accountability, input weekly from the instructor encouraged and motivated.	Health promotion behavior showed a significant difference between pre-test and post test questionnaires. For the change in behaviour most students chose exercise behaviour (40%), following by eating behaviours (20%).

#### Objective 1: Intervention type

Three studies addressed multiple lifestyle behaviours with various interventions. One study employed a combination of education and training modules, personal health tracking, and online goal setting over two years to target smoking, exercise, and nutrition [49]. Another study focused on cardiovascular health with interventions including case-based learning, role-playing, and simulation to cover smoking, exercise, and nutrition [61]. A third study used lectures, group discussions, and videos over 18 weeks to promote general health behaviours [68]. 

Additional studies targeted specific behaviours: two explored exercise and nutrition, with interventions including a nine-week classroom and journal entry program [65] and a 62-hour theoretical plus 22-hour practical health promotion module [52]. Longitudinal and shorter-term studies examined broader health metrics, including an 11-year study tracking blood pressure, weight, and smoking [45] and an eight-week exercise and hydration intervention during clinical placements [56]. Lastly, an e-learning module on smoking cessation and nutrition was compared with an adaptive technology version [55]. 

#### Objective 2: Outcomes

The studies included in this review that included multiple modifiable factors explored three main outcomes relating to behaviour change, knowledge and competence and risk factor identification.

Behavioural modifications were prominently observed [45],4950 [52, 55, 65, 68], particularly regarding smoking cessation and physical activity. In a voluntary lifestyle modification program involving 412 students, smoking cessation was the most notable change, with 114 students quitting smoking and 75 achieving success or transitioning to occasional smoking [45]. Similarly, smoking rates decreased from 18% at T1 to 12% at T3; however, self-reported health showed no significant change, and overall health behaviours declined over time [49]. Physical activity also saw improvement, with one study highlighting regular exercise as the predominant behaviour change reported through thematic analysis of a four-week reflective journal [65]. While there was a positive trend in physical activity [56]both intervention and comparison groups experienced overall weight gain after one year [50]. 

Knowledge and competence improvements were demonstrated in two studies [55, 61]. One study found that both intervention and control groups significantly increased their intention to provide brief counselling, as measured by a validated questionnaire [55]. Another study showed that the intervention group had substantial gains in knowledge, competence, and clinical decision-making, with significant improvements across all validated questionnaire aspects [61]. Finally, Risk factor identification was a key outcome in a large-scale study of 2300 students, revealing that 949 had at least one risk factor. Specifically, 19.8% were smokers, 23.6% had excess bodyweight, and 8.8% had multiple risk factors, this was followed up by an educational intervention module on chronic health conditions and individual behaviour change care plans [45]. 

#### Objective 3: Barriers and facilitators

Across the various studies reviewed, common themes were reported by the studies regarding the implementation and effectiveness of lifestyle behaviour interventions. Despite the diversity in intervention focus, a consistent finding was the need for integrating cardiovascular disease (CVD) modifiable risk teaching into nursing education. Identifying and addressing barriers, such as students’ own lifestyle behaviours and cultural attitudes, as well as recognising facilitators like engaging teaching methods and supportive environments, is crucial for improving outcomes.

Students’ own lifestyle behaviour such as smoking were identified as a barrier to providing interventions to patients [59, 62, 66]. The source of information for students is significant as a potential barrier, with social media a major platform with up to 64% of students seeking nutritional advice there [46, 70]. However, if students rely on unverified accounts, the information may be inaccurate, emphasizing the importance of using evidence-based knowledge. A potential cultural barrier in offering smoking cessation was identified in South Korea, where smoking is a predominantly male behaviour and nurses who are female find this challenging to question [66]. Similarly there is a ‘social taboo’ barrier of young women speaking to older adults about their alcohol use in a Scotland [51]. 

A barrier in personal beliefs with alcohol consumption was highlighted in Scotland, participants identified that as nursing students they should be involved in alcohol brief interventions, but also that people should have the right to use alcohol as they wish in their own home [51]. 

Facilitators for learning were found to have been online or asynchronous intervention delivery as a convenient teaching methodology for students [46, 48, 63]. The inclusion of ‘real life’ scenarios in simulation to help prepare for clinical healthcare interactions [64], and utilisation of a variety of learning techniques to engage active learning [47, 54]. Facilitators for improving lifestyle behaviours were student support for behaviour change from faculty and peers [68] and incorporation of healthy environments at university to support healthy behaviours [49]. 

## Discussion

A paucity of educational interventions inclusive of all four modifiable lifestyle behaviours specifically for CVD risk prevention were identified in this scoping review. The results indicate there are many educational interventions promoting modifiable lifestyle behaviours introduced in pre-registration nursing education, however, the behaviours targeted are majority singular such as smoking. These interventions show limited standardisation in either delivery or content but also identify unique barriers and facilitators to implementation and uptake of modifiable lifestyle behaviour change knowledge, self-efficacy and behaviours.

Delivery of the interventions was most commonly via blended learning methodologies. This aligns with evidence to suggest that a combination of lectures and practice is most effective for learning [72] and there has been found to have a consistent positive effect in comparison with no intervention [73]. It is widely considered best practice to transpose nursing students from passive to active participants in learning experiences [74] therefore traditional didactic methods should be complimented alongside practical elements that can consolidate learning.

Students who undertook simulated practice felt more prepared to engage with patients in clinical practice [63, 67]. The incorporation of active learning methods, such as simulation, peer role-play, and collaborative learning, played a crucial role in enhancing student self-efficacy. These interactive approaches provided students with practical opportunities to apply and reinforce their skills in a controlled environment. By engaging in these activities, students were able to consolidate their learning and build confidence in their clinical abilities. This hands-on experience not only improved their preparedness for real-world patient interactions but also contributed to a deeper understanding of the clinical concepts they had studied.

The use of blended learning was a key component in delivery of effective educational interventions about modifiable risk factors relating to CVD. An educational intervention that utilises blended learning offers a practical and adaptable approach for integrating new content into nursing curricula [75]. Blended learning, which combines online digital resources with traditional face-to-face instruction, provides flexibility and enhances the learning experience by leveraging the strengths of both modalities [75]. This method allows nursing programmes to incorporate essential educational content without significantly disrupting existing schedules or requiring extensive modifications to the curriculum. Research supports the effectiveness of blended learning in improving self-efficacy among students. For example, studies have shown that blended learning interventions lead to notable gains in students’ confidence and competence in various skills [76, 77] This approach fosters a more comprehensive learning environment, contributing to overall improvements in students’ self-efficacy and their ability to apply their knowledge in clinical practice.

The review highlighted various outcomes related to lifestyle behaviour changes. For smoking cessation, one study reported a 5.2% decrease in smoking rates among students following an educational intervention, while another found a 12.9% increase in the intention to quit smoking after a short, one-hour intervention [48, 71]. In terms of nutrition, students were found to consume more calorie-dense fast food and skip meals while at university, suggesting a need for healthier on-campus meal options, cookery lessons, or peer support to address these issues [69, 70] Additionally, 29.3% of students were identified as smokers, either of cigarettes or hookah, and 81% of these had not engaged with the nutritional application used in the study. This finding points to a potential link between smoking and poor dietary choices, as students already participating in physical activity and healthy eating were more likely to use the nutritional tool [70]. This link can be related to Multiple Behaviour Change Research [78] suggesting that that one modifiable lifestyle behaviour that is detrimental such as smoking, co-occurs with a decrease in physical activity and increase in alcohol consumption [79]. The converse is then found if smoking cessation is adopted due to the individual belief processes that adopting multiple healthier lifestyle behaviours will ‘undo’ the harm of smoking. Studies have focused on individual behaviour changes in the general population, with a lack of clarity on combining clusters for the purpose of health promotion and disease prevention. A recent systematic review of the literature [80] focused on chronic disease interventions such as cardiovascular disease and type 2 diabetes mellitus, suggests that targeting more than one behaviour change at a time is effective and more research in this area of multi-level behaviour change is required. Smoking cessation was the only behaviour change that showed no or small effects immediately, suggesting that this behaviour change may require longitudinal follow up and support as positive effects were seen at later time points [81, 82]. The findings of that systematic review also support the results associated with the current scoping review findings. The most prevalent single modifiable lifestyle behaviour for nursing students was smoking, in particular educational interventions on smoking cessation (*n* = 11), followed by interventions that targeted multiple modifible lifestyle behaviours (*n* = 9), only one of which as previously stated was aimed at the four core modifiable lifestyle behaviours for prevention of CVD.

Use of social media to gain knowledge about health and modifiable lifestyle behaviours was a potential perceived barrier to implementing educational interventions [46, 69]. Social media has gained acceptance amongst digital natives in the pre-registration population, specifically millennials and generation Z as a valuable educational resource [83, 84] alongside a source of peer support and an enabler of nursing students to engage in interprofessional education [85]. Despite these positives due to the vast amount of information from diverse sources on social media platforms, there is a heightened vulnerability to misinformation from unreliable sources [86]. Therefore, social media as a learning tool can be used very positively in healthcare education, however, direction towards reputable sources would be required as misinformation can be an issue [87]. 

Cultural or social challenges were highlighted as areas to be considered, such as engaging with sensitive topics like patient smoking status [67], how many units of alcohol are consumed [51] or the fact that the student engages in the behaviour themselves. These are important barriers to consider in relation to the design of an educational intervention as it needs to be socially and culturally appropriate to the setting and population to which it is delivered, or the acceptability will be weakened [88]. 

It is important for nursing students to be aware and informed of CVD as a preventative disease so that they can protect themselves through lifestyle behaviour change and promote the health of those in their care [89]. Further implications from this scoping review are that an educational resource utilising bended learning, comprising the four key modifiable lifestyle behaviours of smoking, physical activity, alcohol consumption and nutrition and directly targeted at CVD risk awareness and behaviour change be developed and embedded in pre-registration curricula.

### Strengths and limitations

This scoping review demonstrates several strengths, particularly its novelty in combining various intervention focuses on modifiable behaviours related to cardiovascular disease prevention in pre-registration nursing education. The review examines educational interventions targeting smoking, physical activity, nutrition, and alcohol consumption, providing a holistic view of efforts to promote healthy lifestyle behaviours among nursing students. Another strength is the international scope of the included studies, with research from North America, Europe, Asia, and the Middle East, offering a global perspective on the topic. The review also employs a rigorous methodology, following accepted frameworks and reporting guidelines, which enhances its credibility and reproducibility.

However, there are some limitations to consider. The review is restricted to English-language publications, potentially missing relevant studies in other languages. Additionally, while the review includes a variety of study designs, the quality of individual studies varies, which may impact the overall strength of the evidence. The focus on pre-registration nursing students also limits the generalisability of findings to other healthcare professions or post-graduate nursing education. The focus of the scoping review on CVD, highlights just one disease, albeit the most prevalent out of the umbrella term of NCDs despite the modifiable lifestyle behaviors being the same to prevent all of them. The conceptual frameworks, models or theories that went into the development of the educational intervention were not extracted, this could be utilised to improve or develop future resources.

The scoping review considered the barriers and the facilitators to the intervention. This is a contentious issue in scoping reviews, that generates lots of discussion but currently is an accepted objective. As such the terms ‘barrier’ and ‘facilitator’ were not included in the search terms for the databases and instead were extracted from the included papers.

Lastly, the heterogeneity of interventions and outcome measures across studies may make it somewhat challenging to draw definitive conclusions about the most effective strategies for promoting modifiable behaviours among nursing students.

## Conclusions

The findings from this scoping review demonstrate that there are several implemented educational interventions that focus on modifiable lifestyle behaviours, aimed at either the pre-registration nurses themselves or the health care service users they will encounter in clinical practice. Overall, these studies indicate that educational interventions have the potential to support positive behavioural change along with improvements in knowledge, confidence, self-efficacy and competence amongst pre-registration nursing students. While these findings are promising, there appears to be a lack of educational interventions that have collectively consider the full range of modifiable behaviours associated with CVD. It is therefore important that this education is embedded into the pre-registration nursing curricula and as early in teaching as possible to empower nursing students and those in their future care.

Future implications following on from the scoping review could see the development of an educational resource, aimed at the population of pre-registration nursing students, focusing on the four modifiable lifestyle behaviours of smoking, alcohol consumption, diet and physical activity in relation to cardiovascular disease prevention or risk reduction. This could be digital, asynchronous, short in duration and include a blended learning strategy that encompasses both active and passive learning methodologies.

## Electronic supplementary material

Below is the link to the electronic supplementary material.

Supplementary Material 1

Supplementary Material 2

Supplementary Material 3

## Data Availability

No datasets were generated or analysed during the current study.

## Abbreviations

CVD: Cardiovascular Disease
WHO: World health organisation
NCD: Non-Communicable Disease
PCC: Population, Concept, Context
ESC: European Society of Cardiology
ACRA: Australian Cardiovascular Health and Rehabilitation Association
MMAT: Mixed Methods Appraisal Tool
