# Supplementary material for: Educational interventions promoting modifiable lifestyle behaviours for cardiovascular disease in pre-registration nursing students: a scoping review
Source: BMC Nurs. 2025 Jul 21;24:952. doi: 10.1186/s12912-025-03611-x (PMC12278611; doi:10.1186/s12912-025-03611-x)
Supplement: Supplementary file 2 — Supplementary Material 2 [file 12912_2025_3611_MOESM2_ESM.pdf]

Author, date	S1	S2	1.1	1.2	1.3	1.4	1.5	2.1	2.2	2.3	2.4	2.5	3.1	3.2	3.3	3.4	3.5	4.1	4.2	4.3	4.4	4.5	5.1	5.2	5.3	5.4	5.5	Total Score	Comments
Butler et al, 2009	Y	Y											1	1	1	1	1											5	
Sohn et al, 2012	Y	Y											1	1	1	1	1											5	4/25 non response=84% response
Shishani et al, 2013	Y	Y											1	1	1	1	1											5	
Schwindt et al, 2016	Y	Y	1	1	1	1	1																					5	
Schwindt et al, 2016	Y	Y						0	1	1	0	1																3	No statement of how randomisation occurred or blinding
Choi et al, 2018	Y	Y											1	1	1	1	1											5	2/23 control group in complete
Hamedeh et al, 2018	Y	Y											1	1	0	1	1											4	60% response rate at phase 3
La Torre et al, 2019	Y	Y											1	1	1	1	1											5	
Zhang et al, 2021	Y	Y											1	1	0	1	1											4	50% response rate phase 2+ 3
Lee and Yunhee, 2022	Y	Y											1	1	1	1	1											5	
Shin and Lee, 2023	Y	Y											1	1	1	1	1											5	
Rabanales Sotos et al, 2015	Y	Y											1	1	1	1	1											5	
O'May et al, 2016	Y	Y	1	1	1	1	1						1	1	0	1	1						1	1	0	0	0	11	Response rate decreased to 65% post
Lavilla-Gracia et al, 2023	Y	Y	1	1	1	1	1						1	1	1	1	1						1	1	1	1	1	15	
Hsaio et al, 2005	Y	Y											1	0	1	1	1											4	content analysis on free text questionnaire questionnaires but not noted as a mixed methods study- aim does not include exploration of rationale
Kara, 2015	Y	Y											1	1	0	1	1											4	percentage response at phases post baseline unclear
McSharry and Timmins, 2016	Y	Y											1	1	1	1	1											5	
Wills and Kelly, 2017	Y	Y											1	1	0	1	0											3	39% response rate phase 3. 3 different interventions therefore ? intervention contamination but volunatry not assigned by researcher
Nevins et al, 2018	Y	Y																0	1	1	0	1						3	Sampling method not outlined. 16.4% post response rate.
Fontaine and Cosette, 2021	Y	Y						1	0	0	0	1																2	researcher not blinded, drop in response rate, unequal groups and attributes
Chang and Chen, 2020	Y	Y	1	1	1	1	1																					5	
Perez-Rivas et al, 2023	Y	Y											1	1	1	1	1											5	
Dong et al, 2023	Y	Y											1	1	1	1	1											5	

Holmberg et al, 2021	Y	Y	0	0	1	1	1	1	1	1	1	1	1	1	1	1	1	13	Qualitative was free-text from the questionnaire- ? descriptive data. no overt methodology
Rosa-Castillo et al, 2022	Y	Y						1	1	0	1	1						4	74.13% response rate- voluntary
Natour and Al-Tell, 2022	Y	Y						1	1	1	1	1						5	
Zaghamir et al, 2023	Y	Y						1	1	1	1	1						5	
Thang et al, 2023	Y	Y						1	1	1	1	1						5	
Mancin et al, 2024	Y	Y						1	1	0	1	1						4	
